# Physicochemical and Antioxidative Properties of Protein Hydrolysates from Residual Goat Placenta Extract by Two Different Methods

**DOI:** 10.3390/foods13203263

**Published:** 2024-10-14

**Authors:** Yinchen Hou, Xinyang Chen, Qihui Shi, Mingyi Zhang, Shengru Yang, Long Pan, Quanping Liu, Yongchao Fan, Rongchao Qiu, Aimei Liao

**Affiliations:** 1 College of Food and Biological Engineering, Henan University of Animal Husbandry and Economy, Zhengzhou 450044, China; 2 Food Laboratory of Zhongyuan, Luohe 462000, China; 3 College of Chemical Engineering, Nanjing Forestry University, Nanjing 210037, China; 4 College of Biological Engineering, Henan University of Technology, Zhengzhou 450001, China

**Keywords:** goat placenta, protein hydrolysates, drying process, physicochemical properties, antioxidant properties

## Abstract

Protein hydrolysates from the goat placenta provide multiple benefits, such as immune system enhancement, antioxidant activities, and reductions in uric acid levels. Despite these benefits, their industrial applications have been underexplored. This study aimed to prepare extract protein hydrolysates (GPERPs) from residual goat placenta extract (GPER) and assess their functional properties, focusing on how different drying methods influence these properties. The essential amino acid contents were 30.94% for the GPER and 34.11% for the GPERPs. Moreover, all the essential amino acids were present, and the amino acid score (AAS) for each exceeded 1.0 in the GPERPs. The foaming properties of the spray-dried GPERPs (95.56 ± 5.89%) were significantly greater than those of the freeze-dried GPERPs (49.13 ± 4.17%) at pH values of 4.0~10.0. The emulsion stability (ES) of the spray-dried GPERPs (453.44 ± 8.13 min) was notably greater than that of the freeze-dried GPERPs (245.58 ± 7.12 min). Furthermore, the water retention capacity (WRC) of the freeze-dried GPERPs (201.49 ± 6.12%) was significantly greater than that of the spray-dried GPERPs (103.35 ± 7.13%), except at pH 10.0 (101.44 ± 8.13%). Similarly, at pH values of 6.0, 8.0, and 10.0, the oil retention capacity (ORC) of the freeze-dried GPERPs (715.58 ± 12.15%) was significantly greater than that of the spray-dried GPERPs (560.56 ± 11.15%), although the opposite trend was noted under acidic conditions. In terms of the antioxidant activity, the ability of the goat placenta extract residual protein hydrolysates (GPERPs) to scavenge DPPH radicals and superoxide anion radicals increased with the increasing peptide powder concentration, and the maximum scavenging rates of the DPPH radicals (39.5 ± 0.56%) and superoxide anions (81.2 ± 0.54%) in the freeze-dried peptide powder were greater than those in the spray-dried peptide powder. These findings contribute to the understanding of the physicochemical and antioxidant properties of GPERPs under various drying methods and provide fundamental data for the development of functional foods based on GPERPs.

## 1. Introduction

The goat placenta is known to contain a variety of bioactive compounds, including growth factors, proteins, peptides, and vitamins, and it facilitates material exchange between the ewe and the fetus during pregnancy. It is particularly abundant in protein (more than 80% of its dry weight), 17 amino acids, 14 trace elements, and other components [1]. In China, the goat placenta is a potent component of Chinese medicine that is widely used as a traditional alternative therapy. The goat placenta is documented in the ancient Chinese medical book *Compendium of Materia Medica* as a traditional tonic [2,3]. Chou et al. [4] found that sheep placenta extract could significantly reduce the aging index, attenuate oxidative stress damage, enhance the antioxidant capacity, and effectively delay aging in mice experiments. Hou et al. [5] conducted an in vitro immunoreactivity assay using goat placental proteins and examined the changes in the immunoreactivity at different temperatures and pH values; the proteins retained their maximal immunoreactivity at 30 °C and demonstrated strong pH adaptability.

Protein hydrolysates, defined as sources of releasable mixtures of bioactive peptides [6], are complex mixtures of oligopeptides, peptides, and free amino acids generated by the enzymatic, chemical, or microbial hydrolysis of whole proteins [7,8]. Active peptides offer significant health benefits, including immune enhancement, cell regeneration, improved skin elasticity, antioxidant effects, and age-delay properties [9,10,11]. Simultaneously, peptides can be used as additives to improve food physicochemical properties, such as the solubility, emulsifying capabilities, thickening abilities, water-holding capacity, and oil-binding abilities, which are crucial for their application in the food, pharmaceutical, and cosmetic industries [12,13,14,15].

The drying methods employed during the production of proteins or protein hydrolysates can significantly impact their functional properties and stability [16,17,18]. Dong et al. [19] found that the drying method significantly influences the physicochemical properties of fish skin protein hydrolysate (SPH), with spray-dried SPH (SPH-SD) exhibiting a higher antioxidant activity and unique structural characteristics compared with freeze-dried SPH (SPH-FD). Soraiyay et al. [20] investigated the effects of spray drying (SD at 180 °C), freeze drying (FD at −35 °C), and foam-mat electrohydrodynamic drying (EHD) on egg white; while the gel hardness and water-holding capacity showed no significant differences, foam-mat EHD produced powders with the highest protein content of 66.1% and a foaming capacity of 725%, closely resembling FD powders in their microstructure and properties. Freeze drying, which is conducted at a low temperature of −30 °C, is widely acclaimed for producing high-quality, high-value products [21]. Zeng et al. [22] employed freeze-drying, spray-drying, and hot-air-drying processes to produce collagen peptide powder from chicken skin, and the freeze-dried collagen peptide powder was better than the hot-air-dried and spray-dried peptide powders in terms of the solubility, emulsion stability, water retention, oil absorption, and water absorption. In a study on the biochemical and emulsification properties of two gluten hydrolysates prepared with the same protease to the same degree of hydrolysis (1.4%) but subjected to different drying processes, E. Linarès et al. [23] reported that while the drying process did not significantly affect the molecular size distribution, hydrophobicity, or solubility of the gluten hydrolysates, the freeze-dried dispersions showed superior emulsifying properties compared with the spray-dried ones, suggesting that the insoluble fraction behavior during drying influences the emulsification performance. Kleekayai et al. [24] compared spray-dried (SD) and freeze-dried (FD) whey protein hydrolysates (WPHs) made with Alcalase^®^ and Prolyve^®^ (Sigma-Aldrich, Dublin, Ireland), finding that the SD-WPHs had higher antioxidative properties due to a greater proportion of peptides (<1 kDa), with the most potent WPH showing oxygen radical absorbance capacity and Trolox equivalent values of 1132 and 686 µmol TE/g, respectively. The application of heat can alter the functional properties of proteins by inducing denaturation and aggregation, which can be mediated by hydrophobic and sulfhydryl–disulfide bond exchange reactions [25]. Protein hydrolysates (peptides, oligopeptides, and amino acids) serve as essential components of food systems, fulfilling both structural and functional roles. They act as structural building blocks, regulating gel properties and enhancing biocompatibility, which are of particular importance for their application in food systems [26]. Consequently, the drying method employed for protein hydrolysates is a critical consideration during the construction of food systems.

Currently, most studies on the goat placenta focus on the preparation and efficacy of peptides and do not investigate the effects of the drying processes on the properties of goat placenta protein hydrolysates [27]. Concurrently, the expansion of the dairy goat industry and the concomitant increase in the goat population have significantly elevated the demand for efficacious goat placenta processing technology [28]. The aim of this study was to investigate the effects of drying methods (freeze drying and spray drying) on the functional properties of GPERPs. Evaluating the effects of different drying methods on the functional properties and stability of protein hydrolysates is essential for optimizing their industrial application. In this study, alterations in the amino acid composition and the distribution of essential amino acids during the reaction process were analyzed. Furthermore, to investigate the potential application of GPERPs in food systems, the foaming performance, emulsification capacity, oil retention capacity, and antioxidative properties of GPERPs under different drying methods were comparatively analyzed. This study aimed to provide a preliminary analysis of how different drying processes influence peptide preparation and their application in food systems.

## 2. Materials and Methods

### 2.1. Materials and Chemicals

Goat placenta was obtained from ewes at parturition and preserved by freezing at −45 °C. The neutral protease (3.97 × 10^3^ U/g) and flavored protease (4.69 × 10^3^ U/L) were purchased from Novozymes (China) Biotechnology Co., Ltd (Tianjin, China). 1,1-Biphenyl-2-pierylhydrazyl (DPPH), Pyrogallol, and 1,10-Phenanthroline were purchased from Beijing Solarbio Science & Technology Co., Ltd. (Beijing, China). All other reagents used in this study were of analytical grade.

### 2.2. Preparation of Protein Hydrolysates from Goat Placenta Extraction Residue

**Figure 1:**
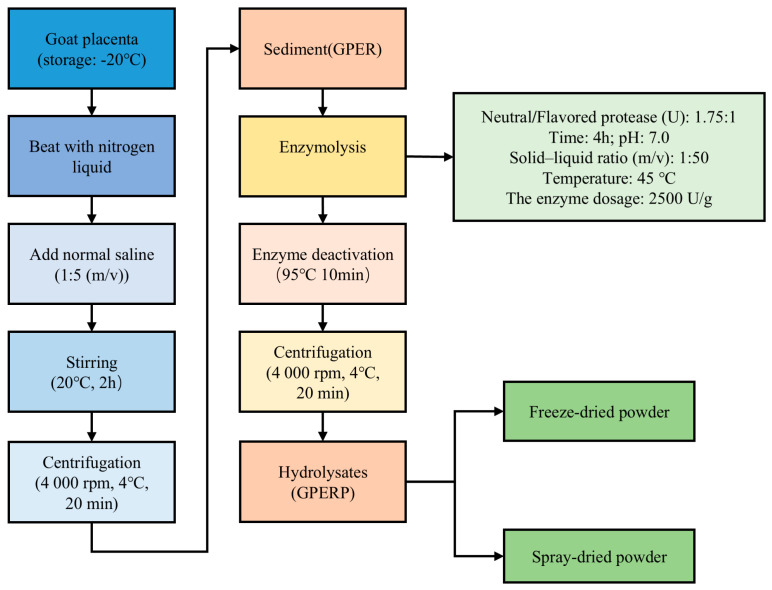
Process for producing protein hydrolysates from goat placenta extraction residue (GPER).

The goat placenta was collected immediately after delivery, frozen at −45 °C with immersion freezing for 12 min, and preserved at −20 °C [29]. The process of producing GPERPs from GPER is illustrated in Figure 1. Two drying methods were employed: freeze drying and spray drying. Both are the most commonly used drying methods for bioactive ingredients in industry [30]. The hydrolysis conditions of the GPERPs were as follows: the enzyme dosage was 2500 U/g; the neutrase and flavorzyme complex ratio was 1.75:1; the hydrolysis time was 4 h; the hydrolysis pH was 7.0; the temperature was 45 °C; and the solid–liquid ratio was 1:50 [31]. The spray-drying process parameters for the inlet air temperature were as follows: 165 ± 1 °C; exhaust air temperature: 98 ± 2 °C; pump speed: 8.0 mL/min; exhaust air pressure: −100~150 Pa; and atomization frequency: 25 Hz. The freeze-drying process parameters for the drying bin temperature were as follows: 30 °C; drying bin pressure: 20 Pa; cold trap temperature: maintained at −50 °C. Under these conditions, the degree of hydrolysis was 39.60%, and the peptide yield was 67.85%.

### 2.3. Analysis of Amino Acid Profiles and Free Amino Acids

An amino acid composition analysis of the GPERPs and GPER was performed using an amino acid analyzer (S433D, Sykam, Germany) [32]. The protein quality was characterized by the amino acid score (AAS). The AAS determines the efficiency with which absorbed dietary nitrogen can meet essential amino acid requirements at safe levels of protein intake. It is calculated as described in FAO/WHO/UNU (2007) [33].

### 2.4. Functional Properties

Among the characteristics of technological ingredients, the foaming properties, emulsifying capacity, and water and oil retention capacities are of the greatest importance in food formulation [34]. The functional properties under different drying methods, including the foaming capacity (FC), foaming stability (FS), water retention capacity (WRC), and oil retention capacity (ORC), were measured.

#### 2.4.1. Foaming Properties

The FC and FS of the hydrolysates were determined as described previously with slight modifications [35]. Aliquots (30 mL) of sample solution (1%, m/v) at various pH values (2.0~10.0) were blended at high speed (10,000 rpm) in a homogenizer (Y50, Shanghai Yuldor Machinery Equipment Co., Ltd., Shanghai, China) for 1 min. The resulting suspensions were rapidly transferred into calibrated tubes, and the total volume of the resultant mixtures was determined after 0.5 and 20 min. The *FC* and *FS* (%) were estimated as follows:

(1) $$\;FC\left(\%\right)=\frac{volume\;after\;whipping\;(0.5\;min)-volume\;before\;whipping}{volume\;before\;whipping}$$

(2) $$FS\left(\%\right)=\frac{volume\;after\;whipping\;(10,30,50,70,90\;min)-volume\;before\;whipping}{volume\;before\;whipping}$$

#### 2.4.2. Emulsifying Properties

The emulsion properties were determined according to the method described by Wang [36]. To prepare the emulsion, 10 mL of soybean oil and 30 mL of sample solution (0.2%, *w*/*v*) at different pH values were shaken together and homogenized at 12,000× *g* and 20 °C for 1 min. A 50 μL sample of the emulsion was taken from the bottom of the container at different times and diluted with 5 mL of a 0.1% (*w*/*v*) sodium dodecyl sulfate (SDS) solution. The absorbance of the diluted emulsion was determined at 500 nm. The emulsifying activity was determined from the absorbance measured immediately after emulsion formation. The emulsifying activity index (*EAI*) and emulsion stability (*ES*) were calculated as follows:

(3) $$EAI(m^{2}/g)=\frac{2\times 2.303\times n\times A}{\rho \times \varphi \times 10000}$$

(4) $$ES\left(min\right)=\frac{A_{0}\times 10}{A_{0}-A_{10min}}$$

where A denotes the absorbance of the emulsion; *n* denotes the dilution ratio; ρ denotes the mass concentration (0.002 g/mL); φ denotes the ratio of the oil phase in the emulsion (0.25); A_0_ denotes the initial absorbance of the emulsion; and A_10min_ is the absorbance of the emulsion after 10 min.

#### 2.4.3. Water Retention Capacity (WRC)

The *WRC* was determined according to the procedure described by Zhang [35]. Briefly, the dried sample (1.0 g) was first mixed with distilled water (20 mL) for 24 h. The mixed sample was subsequently centrifuged (6000 rpm for 15 min) to collect the residue, which was subsequently weighed. The RWC was calculated via Equation (5):

(5) $$WRC\;(g/g)=(W_{2}-W_{1})/W_{1}$$

where *W*_2_ is the weight of the aqueous residue (g) and *W*_1_ is the weight of the dry sample (g).

#### 2.4.4. Oil Retention Capacity (ORC)

The *ORC* analysis was performed as previously described [37]. The sample (1.0 g) was mixed with soybean oil in a centrifuge tube and allowed to stand at room temperature (RT) (25 °C) for 1 h. The mixture was then centrifuged at 1500× *g* for 10 min, the supernatant was poured off, and the solid particles were recovered by filtration. The *ORC* was calculated via Equation (6):

(6) $$ORC(g/g)=(W_{2}-W_{1})/W_{1}$$

where *W*_2_ is the pellet weight (g) and *W*_1_ is the dry weight (g).

### 2.5. Antioxidant Properties

#### 2.5.1. Measurement of Superoxide Radical-Scavenging Capacity

The superoxide radical-scavenging ability of GPERPs treated with different drying methods was tested via the o-triol oxidation method [38], and the Tris-HCl buffer solution and sample solution to be tested were added to different tubes according to the requirements in Table 1. The reaction was terminated by adding 1 mL of 10 mol/L HCl each after 10 min of reaction at room temperature for A_0_, A_1_, A_2_, and A. The absorbance values were measured at 320 nm, and the scavenging rate was determined via Equation (7):

(7) $$O^{2-}\;scavenging\;rate(\%)=1-\frac{A-A_{0}}{A_{1}-A_{2}}\times 100$$

#### 2.5.2. Determination of DPPH Free Radical-Scavenging Capacity

Two milliliters of the sample was mixed with 2 mL of 0.1 mmol/L DPPH–ethanol solution in a test tube and, after standing for 30 min, the absorbance value (A_1_) was determined using anhydrous ethanol as a reference; in the control solution, 2 mL of anhydrous ethanol was used instead of the hydrolyzed solution and, after standing for 30 min, the absorbance value (A_0_) was determined using anhydrous ethanol as a reference; 2 mL of the same concentration of the hydrolyzed solution was mixed with 2 mL of anhydrous ethanol, and the background absorbance value (A_2_) was measured with anhydrous ethanol as a reference [39]. The absorbance values were determined at 517 nm, and the scavenging rate was determined via Equation (8):

(8) $$\mathrm{DPPH}\;scavenging\;rate(\%)=\frac{A_{0}-(A_{1}-A_{2})}{A_{0}}\times 100$$

### 2.6. Statistical Analysis

The results are expressed as the mean ± standard deviation of information obtained via triplicate calculations. Analysis of variance (ANOVA) was performed at a *p* value < 0.05. Multiple comparisons were compared via Duncan’s test.

## 3. Results and Discussion

### 3.1. Preparation of GPER and GPERPs

#### 3.1.1. The Basic Components of the GPER

The basic chemical compositions of the GPER are shown in Table 2. The GPER exhibited a high moisture content (93.12%). On a dry basis, the GPER contained the highest amount of protein in the solid phase at 97.73%. A previous study has shown that freeze-dried and supercritical CO_2_-defatted goat placenta powder contained 91.2% protein [40]. GPER, characterized by its high protein content and low fat content, is well suited for the preparation of bioactive peptides.

#### 3.1.2. Composition and Analysis of Amino Acids

The amino acid composition affects the structure and hydrophobicity of proteins, thereby affecting their biological activity and physicochemical properties [41,42]. The amino acid compositions and amino acid scores (AASs) of the GPER and GPERPs are shown in Table 3 and Table 4. In both the GPER and GPERPs, glutamic acid (Glu), aspartic acid (Asp), and glycine (Gly) were the most abundant amino acids. Ren et al. [43] reported similar results in Tibetan goat placenta peptides, with Glu, Gly, and Asp being the most abundant amino acids. In the GPERPs, the contents of essential amino acids (including threonine (Thr), valine (Val), isoleucine (Ile), phenylalanine (Phe), lysine (Lys), and tryptophan (Trp) were greater than those in the GPER. The process of protein breakdown leads to the formation of amino acids and small peptide chains, thereby increasing the levels of amino acids in the resulting substance. There was minimal difference between the GPER and GPERPs regarding the content of hydrophobic amino acids.

The percentages of essential amino acids in the GPER (30.94%) and GPERPs (34.11%) were compared with the FAO/WHO recommendations. Ren et al. [44] prepared Tibetan goat placenta peptides using a complex enzyme method with papain and neutral protease, with essential amino acids comprising 34.54% of the total amino acids. According to Table 3, all essential amino acids were present, with most having an amino acid score (AAS) equal to or exceeding 1.0. Notably, all the essential amino acid AAS values for the GPERPs were greater than 1, indicating superior nutritional quality compared with the GPER. An AAS greater than 1 indicates that the dietary protein provides the amino acid at levels surpassing the body’s basic requirements, which serves as a positive nutritional indicator, suggesting that this protein offers an advantage in providing essential amino acids [45]. These findings suggest that GPERPs may serve as a valuable nutrient in functional foods, with greater potential for application.

### 3.2. Physicochemical Properties of GPERPs

#### 3.2.1. Foaming Capacity (FC) and Foaming Stability (FS)

The FC and FS represent the increase in the volume of a foam after mixing and the volume remaining over time, respectively. A higher FC requires proteins or peptides to disperse easily in water, migrate quickly to the water–air boundary, and expand to form a protective layer around air bubbles [46]. Numerous factors influence the foaming capacity of proteins or their hydrolysates, including their concentration, molecular weight, ratio of hydrophobic amino acids, and capacity to reduce surface tension [47,48,49]. Proteins with flexible molecules and loose structures generally exhibit better foaming abilities and stabilities than those with rigid structures. The foaming stability is typically highest near the isoelectric point, provided that the solubility remains relatively constant [50].

**Figure 2:**
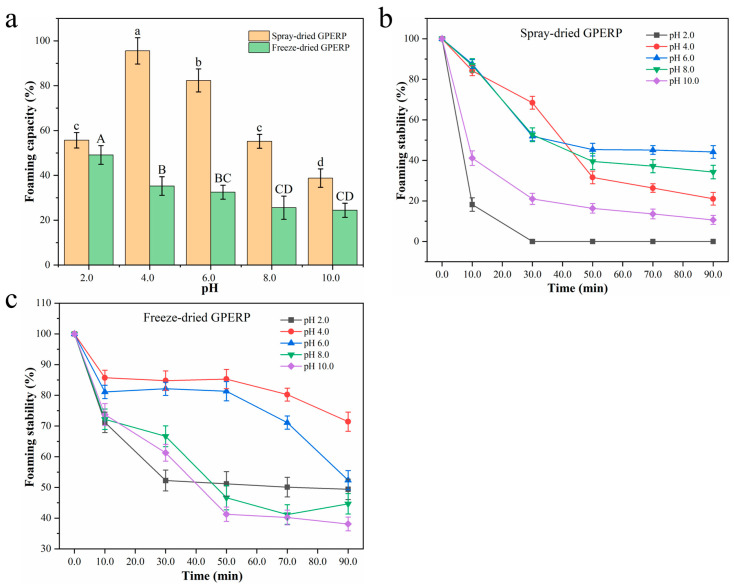
Effects of different drying processes on the foaming characteristics of GPERPs: foaming capacity (**a**), foaming stability of spray-dried GPERPs (**b**), and foaming stability of freeze-dried GPERPs (**c**). Lowercase letters denote comparisons between the foaming capacity of GPERPs under different pH conditions in the spray-dried group, and uppercase letters denote comparisons between the foaming capacity of GPERPs under different pH conditions in the freeze-dried group. Different letters indicate significant differences (*p* < 0.05).

As shown in Figure 2a, the foaming capacity (FC) values of the spray-dried GPERPs were significantly greater than those of the freeze-dried GPERPs across various pH values (*p* < 0.05), except at pH 2.0. At pH 2.0, there was no significant difference in the foaming abilities between the spray-dried and freeze-dried GPERP samples. The inferior foam formation in the freeze-dried GPERPs compared with the spray-dried GPERPs may be attributed to the spherical nature of protein hydrolysates, which hinders their ability to form surface membranes around air bubbles. The foaming properties of GPERPs are more favorable under neutral conditions than under highly acidic or alkaline conditions [51]. Both drying methods yield GPERPs with foaming properties, likely due to the large number of peptides produced during enzymatic hydrolysis. This process reduces the molecular weight and allows more air to enter the molecular interior, increasing the surface activity [52]. Kanwate et al. [53] reported similar results for gelatin extracted from the swim bladder of *Labeo rohita*. As shown in Figure 2b, the FS of the spray-dried GPERPs decreased significantly over time. At both pH 2.0 and pH 10.0, the stability of the spray-dried GPERPs was significantly compromised, as evidenced by the complete absence of foam at pH 2.0 after 30 min and a marked reduction in the foam stability at pH 10, indicating that the FS is markedly influenced by highly acidic or alkaline conditions. The low FS of spray-dried GPERPs may result from conformational changes in the peptide chain, leading to a brittle liquid film that cannot effectively encase air bubbles [54]. As depicted in Figure 2c, the freeze-dried GPERPs exhibited greater stability for the first 50 min within the pH range of 4–6, which may be attributed to the isoelectric point of the peptide. For the foaming properties of goat placenta residue hydrolysates, spray drying clearly offers more advantages.

#### 3.2.2. Emulsifying Activity Index (EAI) and Emulsion Stability (ES)

**Figure 3:**
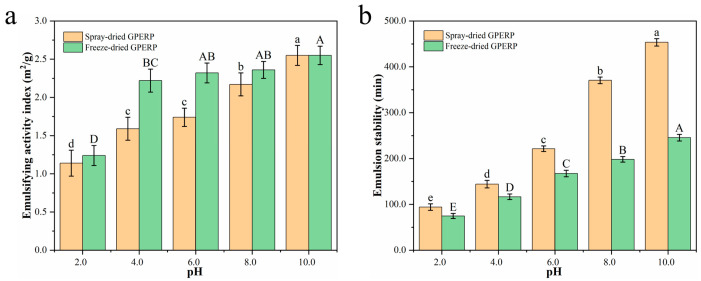
Effect of spray and freeze drying on the emulsifying properties of GPERPs: emulsifying activity index (**a**) and emulsifying stability (**b**). Lowercase letters denote comparisons between the emulsifying activity index and emulsion stability of GPERPs under different pH conditions in the spray-dried group, and uppercase letters denote comparisons between the emulsifying activity index and emulsion stability of GPERPs under different pH conditions in the freeze-dried group. Different letters indicate significant differences (*p* < 0.05).

Peptides present in self-assembled gel systems, and Pickering emulsions can modulate the microstructures and overall properties of both emulsions and gels because of their distinct structural characteristics and hydrophilic/hydrophobic properties. Moreover, peptides exhibit remarkable surface activity and interfacial stability within these systems. The emulsification activity indexes (EAIs) and emulsion stabilities (ESs) of the spray-dried and freeze-dried GPERPs are shown in Figure 3a,b. The EAIs and ESs of the goat placenta protein hydrolysates from the two drying methods showed different trends. Among them, the EAI of the freeze-dried samples was significantly greater than that of the spray-dried samples, whereas the emulsification stability exhibited the opposite trend. The EAI of the GPERPs increased with the increasing pH (*p* < 0.05); at pH values of 4.0 and 6.0, the EAI of the freeze-dried GPERPs was significantly greater than that of the spray-dried GPERPs, whereas at pH values of 8.0 and 10.0, there was no significant difference in the EAI. At lower pH values, the hydrolysate has a positive charge, causing electrostatic repulsion between molecules and hindering the formation of an emulsification system. Under alkaline conditions, the hydrolysate promotes oil and water interface diffusion, resulting in effective emulsification [28]. The impact of the pH on the ES of the GPERPs is depicted in Figure 3b. Both the spray-dried and freeze-dried GPERPs exhibited increased ESs with the increasing pH (*p* < 0.05). The ES for the spray-dried GPERPs was 94.13 min at pH 2.0, which significantly increased to 453.44 min at pH 10. Similarly, the freeze-dried GPERPs showed a significant increase in their ES (*p* < 0.05), from 74.67 min at pH 2.0 to 245.58 min at pH 10.0.

The presence of more hydrophobic amino acids may be linked to the observed effects. Du et al. [55] performed a functional characterization of freeze-dried, vacuum-dried, and spray-dried egg white peptide powders and reported that the emulsifications and emulsion stabilities of the vacuum-dried and spray-dried samples were significantly lower than those of the freeze-dried samples. During spray drying and heating, protein molecules re-aggregate through hydrogen and disulfide bonds, reducing their flexibility and significantly impacting their emulsification and emulsification stability. Proteins rich in hydrophobic amino acids within the emulsion–gel matrix can enhance both the stability and emulsification characteristics [56,57].

#### 3.2.3. Water Retention Capacity (WRC) and Oil Retention Capacity (ORC)

The water retention capacity refers to the ability of proteins or their hydrolysates to absorb water at room temperature, which is affected by the pH, temperature, surface charge, ionic strength, protein structure, and amino acid composition [58]. For proteins to exhibit a good water retention capacity, three conditions must be met: protein or hydrolysate particles fully swell after rehydration but do not dissolve; protein or hydrolysate particles have good viscosity after rehydration; and proteins or hydrolysates form gel network structures [59].

**Figure 4:**
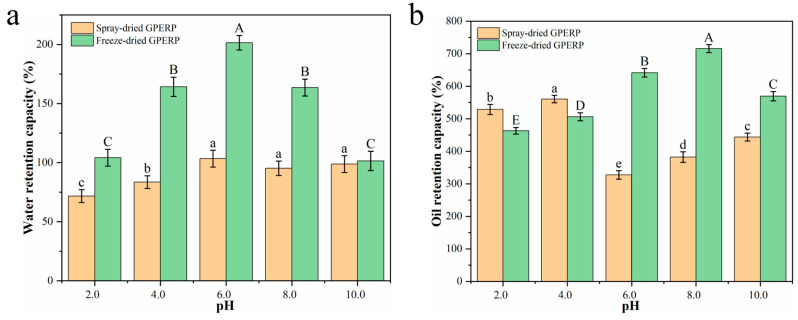
Effects of spray and freeze drying on the water and oil retention capacities of GPERPs: water retention capacity (**a**) and oil retention capacity (**b**). Lowercase letters denote comparisons between the water and oil retention capacities of GPERPs under different pH conditions in the spray-dried group, and uppercase letters denote comparisons between the water and oil retention capacities of GPERPs under different pH conditions in the freeze-dried group. Different letters indicate significant differences (*p* < 0.05).

The effects of different pH values on the WRC of the GPERPs are shown in Figure 4a. The WRC of the freeze-dried GPERPs was significantly greater than that of the spray-dried GPERPs (*p* < 0.05), except at pH 10.0. The WRC of the freeze-dried GPERPs reached its maximum value of 201.49% when the pH was 6. At pH values between 6.0 and 8.0, the WRCs of the spray-dried GPERP samples were not significantly different but were significantly greater than those at pH 2.0 and 4.0 (*p* < 0.05). Overall, the WRC of the freeze-dried GPERPs was greater than that of the spray-dried GPERPs, possibly because the spray-dried WGPERP particles were fine, dissolved quickly in water, and had difficulty swelling, whereas the freeze-dried powder particles were coarse, had poor solubility, and swelled easily, so the WRC was greater than that of the spray-dried WGPERPs.

The oil retention capacity (ORC) is another crucial functional property of proteins or hydrolysates in food systems. The lipophilic properties of proteins are related to the nature of the lipophilic groups on the surfaces of their molecules [60]. The nonpolar amino acid side chains of proteins can form hydrophobic interactions with the hydrocarbon chains of lipids, which influence their oil-binding capacity [61]. The effect of the pH on the ORC of the GPERPs is shown in Figure 4b. The figure indicates that the ORC of the spray-dried GPERPs under acidic conditions exceeds that under alkaline conditions, with a maximum ORC of 560% at pH 4.0. Compared with that of the spray-dried GPERPs, the ORC of the freeze-dried GPERPs exhibited the opposite trend. Under alkaline conditions (pH 8.0), the ORC of the freeze-dried GPERPs was 715%, which was significantly greater than that of the spray-dried GPERPs (382%) (*p* < 0.05).

### 3.3. Antioxidant Properties of GPERPs

Antioxidant peptides can prevent oxidation by reacting with free radicals, transforming them into more stable products, and aborting the chain reaction of free radicals. O^2−^ is the primary reactive oxygen radical in living organisms; although it is not highly active, it can produce H_2_O_2_ and hydroxyl radicals through disproportionation reactions and other pathways. Many studies have shown that peptides effectively scavenge free radicals and that the scavenging effect of peptides on free radicals is closely related to the amino acids they contain [62,63]. 

**Figure 5:**
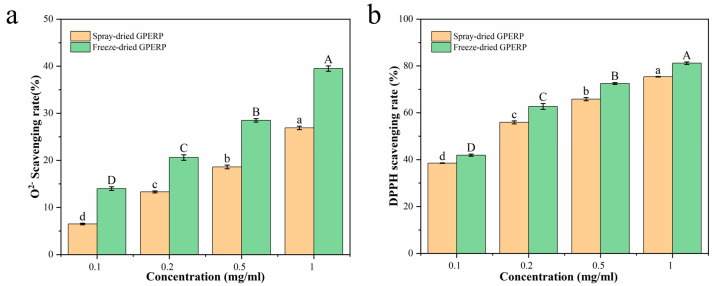
Effects of spray and freeze drying on the antioxidant properties of GPERPs: O^2−^ free radicals (**a**) and DPPH free radicals (**b**). Lowercase letters denote comparisons between the O^2−^ or DPPH free radicals of GPERPs at different concentrations in the spray-dried group, and uppercase letters denote comparisons between the O^2−^ or DPPH free radicals of GPERPs at different concentrations in the freeze-dried group. Different letters indicate significant differences (*p* < 0.05).

The O^2−^-scavenging effects of the different concentrations of the goat placenta peptide powders obtained via the two drying methods are shown in Figure 5a. The O^2−^-scavenging ability of the peptide powder is related to its solution concentration; as the concentration increases, its scavenging ability improves for peptide powders produced via different processes. At the same concentration, the magnitude of the O^2−^-scavenging rate was as follows: that of the freeze-dried peptide powder was greater than that of the spray-dried peptide powder. Certain factors inherent to the spray-drying process—including the shear force of the nozzle, the thermal stress generated during droplet drying, and the adsorption of proteins or peptides at the air–liquid interface—can potentially damage these biomolecules [64]. The DPPH-scavenging activities of the placental peptides obtained via different drying methods are shown in Figure 5b. The DPPH-scavenging activity of the peptides obtained via freeze drying at the same concentration was slightly greater than that of the peptides obtained via spray drying. DPPH, a stable free radical with a single electron, participates in oxidation reactions. After the addition of free radical scavengers, the DPPH lone pair of electrons is paired, resulting in a decrease in the absorbance value of its ethanol solution at a wavelength of 517 nm, and the degree of discoloration is quantitatively related to the number of electrons it accepts. Wang et al. demonstrated that the spray-drying parameters also influence the antioxidant capacity of peptide powder. These findings indicate that with an increasing inlet temperature, the antioxidant capacity decreases. However, up to a certain threshold, an increased feed rate can positively affect the antioxidant capacity, possibly by safeguarding heat-sensitive components [65,66]. Guo et al. [67] reported that the presence of amino acids such as Try, Phe, and Lys at the C or N terminus of a peptide exhibits strong antioxidant activity. However, it is not only the presence of some favorable amino acids in the peptide sequence that is crucial for the activity of the peptide but also their correct position in the peptide sequence. However, we do not know the exact position of these amino acids in goat placenta peptides. Therefore, determining the sequence of the antioxidant peptides is the direction of future research, which can be facilitated by further purification of the peptides by ultrafiltration to facilitate the discovery.

## 4. Conclusions

Studying the changes in the physicochemical properties of GPERPs under different drying conditions is essential for understanding them and applying them in food systems. From the investigations of the present work, it can be concluded that the different drying methods (freeze drying and spray drying) of the GPERPs had an impact on the physicochemical and antioxidant properties. Moreover, GPERPs contain more essential amino acids than GPER, and the AAS values of all the essential amino acids of GPERPs are greater than 1, indicating that its nutritional quality is superior to that of GPER. The functional property results revealed that the spray-dried GPERPs have superior foaming properties and emulsifying activity, whereas freeze-dried GPERPs have a better water retention capacity, and oil retention capacity at most pH values. GPERPs have good antioxidant properties, and freeze-dried powder generally outperforms spray-dried peptide powder. Overall, freeze drying or spray drying could be appropriate drying methods for the preparation of hydrolysates from residual goat placenta extract with better functionalities. The findings of this study will enhance our understanding of the functional properties of GPERPs, guiding their application in self-assembly, Pickering colloids, and other food ingredients, thereby expanding the use of goat placenta residues and increasing the economic value of this byproduct.

## Figures and Tables

**Table 1:** The amount of reagent added was used to determine the superoxide radical-scavenging capacity.

	Tris-HCl (50 mM)	Sample (50 g/L)	HCl (10 M)	Catechoroglucinol (3 mM)
A_0_	5 mL	1 mL	1 mL	1 mL
A	5 mL	1 mL	-	1 mL
A_1_	5 mL	-	-	1 mL
A_2_	5 mL	-	1 mL	1 mL

**Table 2:** Proximate composition of the extracted goat placenta residue.

Composition	Content (%)
Moisture	93.121 ± 0.761
Protein	6.723 ± 0.213
Fat	0.062 ± 0.005
Total Carbohydrates	0.017 ± 0.002
Ash	0.023 ± 0.009

**Table 3:** Amino acid composition of GPER and GPERPs.

Amino Acid	GPER/%	GPERPs/%
Asp	8.59	7.88
*Thr	4.18	4.61
Ser	4.41	3.94
Glu	13.41	11.64
Ala	6.83	6.97
Cys	1.85	1.79
*Val	4.62	5.31
Met	2.14	2.10
*Ile	3.69	4.29
*Leu	7.15	6.97
Tyr	3.08	4.34
*Phe	3.86	4.26
*Lys	6.30	6.92
His	2.10	2.70
Arg	7.40	7.66
Pro	7.09	6.42
Gly	12.46	11.65
*Trp	1.14	1.75
Essential amino acids	30.94	34.11
Hydrophobic amino acids	45.7	45.87

Note: * represents essential amino acids.

**Table 4:** Essential amino acid compositions (AASs) of GPER and GPERPs.

Amino Acid	FAO/WHO	GPER		GPERPs	
		Content	AAS	Content	AAS
Ile	40	36.9	0.92	42.9	1.07
Leu	70	71.5	1.02	69.7	1.00
Lys	55	63.0	1.15	69.2	1.26
Met + Cys	35	39.9	1.14	38.9	1.11
Phe + Tyr	60	69.4	1.16	86.0	1.43
Thr	40	41.8	1.05	46.1	1.15
Trp	10	11.4	1.14	17.5	1.75
Val	50	46.2	0.92	53.1	1.06
Value	360	380.1		423.4	

Note: content represents the amount (mg) of essential amino acids per gram of protein.

## Data Availability

The datasets generated for this study are available upon request to the first author.
